# Occipital meningoencephalocele in a newborn: A case report in East Africa

**DOI:** 10.1016/j.ijscr.2024.110058

**Published:** 2024-07-20

**Authors:** Elisamia Ngowi, Mugisha Clement Mazoko, Zainab Fidaali, Pilly Ally, Yaser Abdallah

**Affiliations:** a Department of Paediatrics and Child Health, Aga Khan Hospital Tanzania, P.O. Box 2289, Dar Es Salaam, Tanzania; b Department of Paediatrics and Child Health, Aga Khan University, P.O. Box 38129, Dar Es Salaam, Tanzania; c Department of Radiology, Aga Khan Hospital, P.O. Box 2289, Dar Es Salaam, Tanzania; d Department of Surgery, Aga Khan Hospital, P.O. Box 2289, Dar Es Salaam, Tanzania; e Department of Surgery, Aga Khan University, P.O. Box 38129, Dar Es Salaam, Tanzania

**Keywords:** Meningoencephalocele, Encephalocele, Case Report

## Abstract

**Introduction:**

Encephalocele refers to protrusion of cranial contents through a bony skull defect. Prevalence of encephaloceles in East Africa is approximately 2 per 10,000 livebirths, with occipital encephaloceles making the least proportion of these in this region. We present a case which was diagnosed postnatally and managed surgically with good outcome and few anticipated complications.

**Case presentation:**

Newborn baby delivered to a 26-year-old mother at 38 weeks of gestation by spontaneous vaginal delivery, with swelling on the occipital region since birth. Physical examination revealed a mass measuring 8 cm by 6 cm over the occiput. Initial cranial ultrasound and MRI of the brain revealed an occipital myelomeningocele with part of the right cerebellar lobe, meninges, and CSF herniating through the defect in the occipital skull bone. Surgical correction was successfully done. The patient developed CSF leakage due to hydrocephalus 1-week post-surgery and VP shunt placed to relieve the increased intracranial pressure.

**Discussion:**

This case highlights a very rare neurosurgical congenital defect in East Africa that was managed as early as possible in a low resource setting with minimal post-surgical complications.

**Conclusion:**

There is a need for high index of suspicion for encephalocele during antenatal ultrasound screening for prenatal diagnosis. Early surgical repair and prompt post operative follow up help to minimize complications especially in low resource settings where morbidity can be high due to high costs of managing complications.

## Highlights

• Occipital encephaloceles are the least common encephaloceles in Eastern Africa
• Prenatal anomaly scans are important to diagnose the condition and prepare parents for post-delivery management plans.
• Hydrocephalus is a common post-surgical complication that should be anticipated by prompt.
• Surgical Repair in low resource settings can be achieved with good outcomes

## 1 Introduction

Encephalocele is a rare congenital bony defect that results in displacement of cranial contents out of the normal boundaries of the skull. The contents displaced may include meninges alone (meningocele), meninges with brain tissue (meningoencephalocele) and when linked with ventricles (meningoencephalocystocele). Encephaloceles can occur basally, occipitally, anteriorly (sincipital) or can be convex [1].

Worldwide, there are about 18 cases of neurotube defects (NTD) per 10,000 livebirths, and only 1 in 10,000 livebirths will be affected by congenital encephalocele [2]. A meta-analysis in eastern Africa revealed a pooled prevalence of 33 NTDs per 10,000 births, with encephalocele being the rarest at 2.33 per 10,000 births [3]. There is a regional difference in prevalence of occipital encephaloceles, with higher numbers reported in North America, while anterior encephaloceles are more common in other regions on the world like Asia and Africa. Females are more likely to present with congenital occipital encephalocele than males [4].

The exact mechanism for the development encephaloceles is unknown but is pointed to a multifactorial origin. Theories exist explaining failure of closure of the neural tube and membranous calvarium, and failure of ossification or induction of ossification due to abnormal neural tube closure. Occipital encephaloceles are postulated to result from failure of normal closure of primary neural tube [5]. Symptoms of encephalocele are dependent on the size, location and amount of brain tissue protruding from the skull. Occipital encephaloceles are often associated with neurological complications whereas anterior encephaloceles have better prognosis because they usually do not contain brain tissue [6].

Risk factors associated with congenital encephalocele include maternal infections during pregnancy, consanguinity, and low serum levels of vitamin B_12_ and/or folic acid in pregnant women in the first trimester. Prevention can be done by folic acid supplementation during preconception period [7,8].

Diagnosis can be made prenatally by ultrasound imaging and maternal serum alpha-fetoprotein levels. Postnatal, diagnosis is by clinical examination of the patient's swelling and radiological confirmation of the contents together with bone abnormalities. A computerised tomography scan (CT scan) with three-dimensional reconstruction is a preferred diagnostic modality to visualize internal and external bone abnormalities while Magnetic resonance imaging can reveal contents of the encephalocele and other brain anomalies [8,9]. These imaging modalities provide guidance to the surgeon for repair, as they delineate the bony defect and associated anomalies [10].

Occipital encephalocele is managed by surgical repair preferably before 4 months of age, the earlier the better. A multidisciplinary approach is necessary to ensure there are no or minimal long term complications post repair. The aim of surgery is to achieve a watertight intradural closure, resection of sac and reconstruction of the bony defect. Most common post-surgical complications include Cerebrospinal fluid (CSF) leakage, hydrocephalus and meningitis [10].

Prognosis depends on the size of the sac, neural tissue content, hydrocephaly, infection and associated pathologies or anomalies accompanying the occipital encephalocele. Early and prompt neurosurgical intervention with appropriate pre and post operative care are important determinants of reducing complications [11].

We present a case of occipital encephalocele, surgically managed in a low resource country with post-surgical complications of CSF leakage and hydrocephalus. The aim of the case report is to highlight the importance of early surgical repair with watertight suture to avoid CSF leakage and meningitis. We also stress the need for radiologist to consider this anomaly during antenatal ultrasound screening to anticipate occipital encephaloceles in our setting.

This case report has been reported in line with the SCARE criteria [12].

## 2 Case presentation

We present a case of a female baby, born at 38 weeks of gestation by spontaneous vaginal delivery to a 26-year-old mother. This is the first born. Antenatal history was unremarkable. The mother had negative serological test results of HIV, syphilis, and hepatitis. Antenatal ultrasound was normal. Liquor was meconium stained. The baby had an APGAR Score of 6 and 8 in the 1st and 5th minute of life after birth. Birthweight was 3.2 kg, body length of 49 cm and occipital frontal circumference of 32.5 cm which were appropriate for age.

On examination at birth, had a mass on the occiput, soft, immobile, non-tender, measuring 8 cm by 6 cm in widest diameters and protruding by about 3 cm off the scalp. Was generally pink with no facial or other skeletal dysmorphic features. Neurologically, sucking, Moro and grasp reflexes were weak and had poor tone on vertical and ventral suspension. The baby developed fast breathing with features of respiratory distress such as grunting and lower chest indrawing within the first 4 h of life. The baby was kept on Continuous positive airway pressure for respiratory support as symptoms resolved within 24 h. Cardiovascular examination and other systems were normal on examination.

Investigations revealed leucocytes (white blood cell count) of 14.56 × 10^9^: normal <26 × 10^9^, with haemoglobin count of 19.3 g/dL: normal >15.2 g/dL, platelet count 348: normal 150–450, a C-reactive protein concentration of 0.01 mg/L (reference range < 5 mg/dL), Serum Total Bilirubin of 256 μmol/L (which was within the cut off for phototherapy for age, that is >240). The baby was kept under phototherapy according to serum bilirubin levels and withdrawn after subsequent serum bilirubin levels of 212 μmol/L, which is below the treatment threshold.

**Fig. 1:**
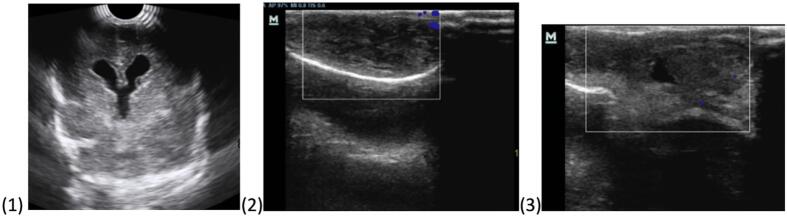
Cranial ultrasound revealed dilated lateral ventricles with a soft tissue mass with some fluid content at the occipital lobe in continuation with the underlying brain through a defect in the skull bone.

**Fig. 2:**
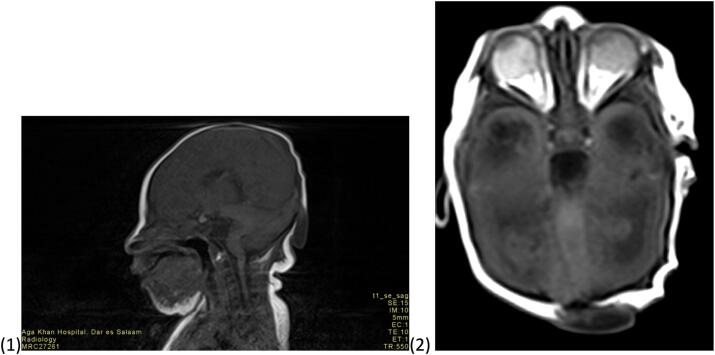
Multiplanar multisequence MRI was performed on the brain in standard orthogonal planes. MRI brain T1 sagittal and axial images showing the occipital encephalomeningocele with small part of right cerebellar lobe, meninges and CSF herniating into it.

Cranial Ultrasound was done, with findings suggestive of occipital meningoencephalocele with mild ventriculomegaly Fig. 1. Magnetic resonance imaging (MRI) was done following the cranial ultrasound. MRI showed features of occipital encephalomeningocele with small part of right cerebella lobe, meninges, and cerebrospinal fluid herniating into it Fig. 2.

The child was reviewed by a neurosurgeon and decision for surgical intervention was made. Baby was under general anaesthesia in prone position, head held on a horseshoe Mayfield holder. An elliptical incision was made about 5 cm from the root of the meningoencephalocele, subcutaneous dissection was done to the level of the skull and dura identified, the skin covering the opening was removed to visualize the content. There was redundant atrophic brain matter, but no big vessels identified. Cauterization was done with bipolar diathermy and redundant extra cranial brain resected.

**Fig. 3:**
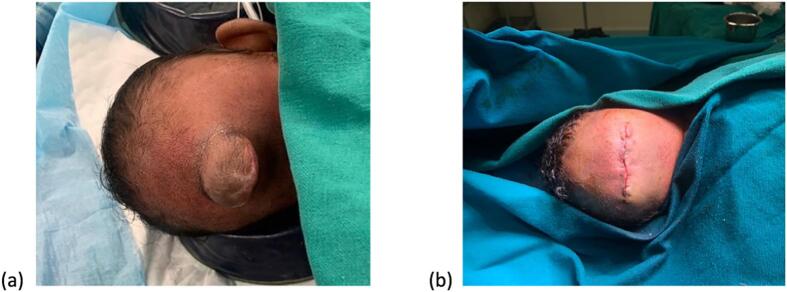
Pre-operative (a) and post-operative (b) images of the baby's occipital lesion.

Dura trimmed and repair of the dura done with nylon 4–0, pericranium also mobilized and repaired the 2.5 cm defect. The skin closed in 2 layer with vicryl 3–0 and nylon-3-0. Valsalva manoeuvre was done and there was no CSF leak Fig. 3. CSF was taken for laboratory tests. The CSF was turbid in appearance with no white blood cells, no red blood cells, protein of 229 mg/dL (normal rage: 15–45 mg/dL), no organism seen on gram stain and AFB Stain. The CSF culture did not grow any pathogen.

**Fig. 4:**
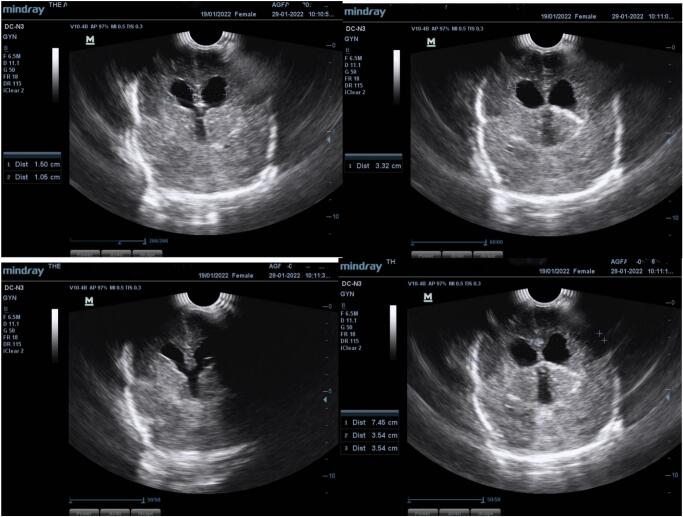
Cranial ultrasound scan done 1 week post meningoencephalocele repair: The lateral, third and fourth ventricles are dilated, the dilatation has significantly increased since the previous scan. There are subtle bilateral cortical hypoechoic areas affecting the fronto-temporal region. The paraventricular regions are normal. There is diffuse lack of sulci and gyri.

**Fig. 5:**
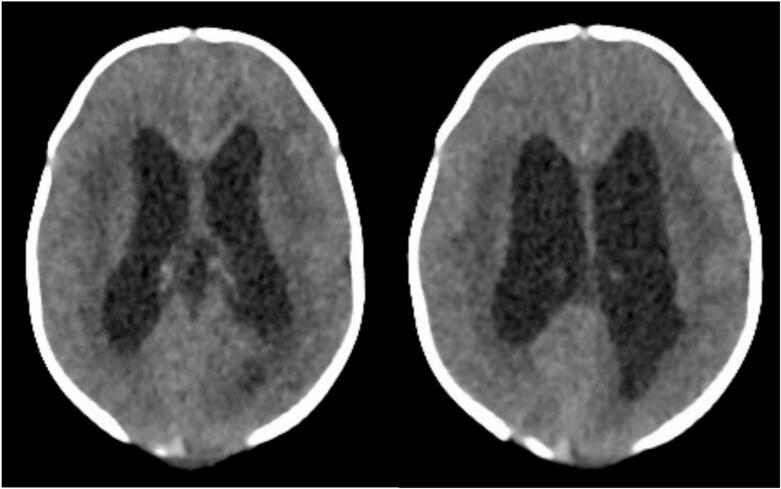
Non-contrasted volumetric CT imaging of the head from the base of skull to the vertex viewed in appropriate reformats and window settings: Moderately dilated lateral and third ventricle (severity has increased in comparison to previous MRI of brain). The fourth ventricle is not dilated. The occipital bone defect is noted with only fat contents within the herniated sac, no brain tissue seen. Persistent diffuse loss of sulcations noted. Corpus callosum not clearly defined.

The child developed CSF leakage at the suture site, 1-week post-surgery. Cranial ultrasound was done with showed features suggestive of communicating hydrocephalus Fig. 4. A computed tomography (CT) Scan was done which revealed moderate hydrocephalus Fig. 5. A ventriculoperitoneal (VP) shunt was placed by the neurosurgeon successfully and CSF samples collected for laboratory investigations. CSF was clear in appearance with no white blood cells, red blood cells, protein was 28 mg/dL (normal range: 15–45 mg/dL) and no organism seen on Gram stain and AFB Stain. It should be noted that the Meningoencephalocele repair was done on day 5 of life while the VP shunt was on day 8 after meningoencephalocele repair. Post operative care was offered as per hospital protocols and the baby was discharged 3 days later.

During follow-up clinic visits, the infant was noted to have hypertonicity of upper and lower limbs and was initiated with regular physiotherapy and occupational therapy sessions for neurodevelopmental rehabilitation to improve head and neck control, spine movement and reduce hypertonicity.

## 3 Discussion

Cephalocele refers to protrusion of meninges with or without brain tissue through a skull defect. Cephaloceles can also be occipital-cervical, parietal, lateral, interfrontal, temporal, spheno-orbital, spheno-maxillary and nasopharyngeal. An encephalocele is protrusion of skull contents through a bony defect. Occipital encephalocele is when the herniated sac protrudes through the occipital bone. Other subcategories of encephaloceles include basal, convexity and sincipital encephalocele, dependent on the part of skull bone the sac has herniated. The sac contents determine the subtype of encephalocele. Our patient had an occipital meningoencephalocele given that the herniated sac contained meninges with brain tissue as described in the MRI. [1].

According to different studies published, the general prevalence of encephalocele is about 1 in 5000 to 10,000 livebirths worldwide. In East Africa, highest prevalence in the region was reported in Ethiopia (60 per 1000 births) compared to other countries like Tanzania where the index patient was diagnosed [3]. A study at the national referral hospital in Dar es salaam, Tanzania, showed an incidence of encephalocele of 0.29 per 1000 births between the years 2000 and 2002 [13].

The specific etiopathogenesis of encephaloceles is not known, but there are several suggested theories. Maternal infections, maternal hyperthermia, deficiency in folic acid and vitamin B_12_, hypervitaminosis A, during the first trimester are sought to be associated with higher risks for newborn encephaloceles. Female gender has been associated with a higher incidence than males. Genetic predisposition has been suggested with higher risks in consanguinity than nonconsanguineous parents. Genes such as Centrosomal protein 290 (CEP290) have been linked with encephaloceles [7,8,14]. Our patient was female, identifying the higher risk for gender, but did not identify any other specific risks as suggested, and there was no reported consanguinity although we cannot completely rule out consanguinity as genetic tests on parents were not done. Encephaloceles are also associated with other neurodevelopmental anomalies like Dandy-walker syndrome and Chiari malformations [7], however, our patient did not present with dysmorphic features of characteristic for any syndrome.

Diagnosis can be made prenatally or postnatally. Prenatal diagnosis relies on radiological findings, with ultrasonography as the most extensively applied modality. Ultrasonography can be affected by amniotic fluid and mother's anatomical habitus, but nevertheless, it's been shown to detect at least 80 % of foetuses with occipital encephalocele. CT scan with three-dimensional reconstruction is not recommended before birth due to possible effects of radiation, however it is of value postnatally due to its excellent delineation of bony defects. MRI is preferred for visualising the soft tissue components of the encephalocele, including T1 and T2-weighted sequences that can depict multiple projections of brain tissue, cranial skull defects and the extent of cerebral tissue that has herniated into the sac. MRI with angiography can provide extra benefit in depicting relationship of the herniated sac with venous sinuses [15]. Our case was diagnosed post natally after physical examination findings of the occipital mass and radiological imaging confirmation of the lesion. The patient presented at our facility two days before delivery for a late obstetrics ultrasound scan and had been attending antenatal clinic in another centre and we could not access previous antenatal scans. This explains why the anomaly was undiagnosed until birth. Antenatal diagnosis provides ground for anticipatory management and preparations for corrective surgery.

Management of encephaloceles is by surgical correction. Occipital encephalocele pose numerous challenges depending on their sizes. Sac contents are assessed for viability and placed in their anatomical positions accordingly. Goal of surgery is to achieve a water-tight suture after repair to avoid leakage of CSF and ascending of infection which can cause meningitis. In some case series, VP shunt is successfully placed before repair of the encephalocele [16,17]. Cranioplasty is deployed in larger defects to achieve good cosmetic outcomes. Materials used for cranioplasty can include, but not limited to, methyl methacrylate, demineralized bone matrix and titanium meshes [10]. Our patient did not have a giant occipital encephalocele, hence cranioplasty was not done. However, the patient developed CSF leakage, probably due to the high pressure from the hydrocephalus that overcame the suture strength.

CSF leakage is the common post operative complications reported after repair of encephaloceles with other complications including meningitis, surgical site infection and cosmetic derangement [7]. Our patient presented with CSF leakage one-week post-surgery and increased intracranial pressure manifesting as hydrocephalus. However, there was no evidence of CSF infection or clinical signs and symptoms of meningitis. Ventriculo-peritoneal shunt was placed to relieve the increased intracranial pressure. Cosmetically, our patient was not attended by plastic surgeon due to unavailability at the time in our centre. However, the surgical site was sutured satisfactorily by the attending surgeon. Although CSF protein was elevated, there was no evidence of meningitis. The elevation in CSF protein could be because of inflammation caused by the surgery in addition to leakage through the defect [18].

Occipital encephaloceles require considerable attention as they're in closest proximity to brainstem, cerebellum, and visual cortex than other types of encephaloceles. They have higher incidence of complicating to hydrocephalus, hence frequent follow up is paramount with radiological screening [7]. Our patient was screened during the first follow up for hydrocephalus and VP shunt placed immediately.

Mortality rate has been reported in some case series to be between 29 and 33 %, mostly associated with post operative infection [11]. Morbidity from occipital encephalocele is mostly due to development of hydrocephalus, seizure disorder, corpus callosal abnormalities and microcephaly. There has been differing reports about location of encephalocele predicting the long-term outcome, with some studies reporting no association between location and outcome. It has been demonstrated that around 48 % of children will have adequate development post-surgical repair compared to 25 % having severe impairment. Occipital and parietal encephaloceles have a worse prognosis than other types [19]. Development of hydrocephalus and presence of associated intracranial abnormalities are predictors of bad outcome, specifically developmental delay. Seizure disorder as well as microcephaly are also associated with poor outcome [19]. Our patient did not present with seizures but presented with hydrocephalus which was intervened immediately. Developmental delay was seen with our patient as delayed neck control, but also slight stiffening of limbs.

Currently, the child is on regular physiotherapy and occupational therapy follow up, in addition to regular paediatric clinic visits for follow up on general growth and development.

## 4 Conclusions

Physicians' ought to emphasize anomaly scans in second trimester to pick encephaloceles early, aiding in anticipatory management plans as well as counselling the parents on the condition.

## Consent for publication

Written informed consent was obtained from the patient's legal guardian (mother) for publication of this case report and the accompanying images. A copy of the written consent is available for review by the corresponding author of this journal.

## Ethical approval

Not required for case reports at our hospital for single case reports.

## Funding

No funds were needed to publish this case.

## Guarantor

Elisamia Ngowi is the main guarantor of this research work.

## Research registration number

This case report is not a First in Man study.

## CRediT authorship contribution statement

Elisamia Ngowi was involved in the conception, study design, acquisition, and interpretation of data, and drafting of the manuscript.
Zainab Fidaali and Pilly Ally were involved in interpretation of radiological data and reviewed the literature of the research work.
Yaser Abdallah was involved in interpretation of clinical data and literature review.
Mugisha Clement Mazoko supervised and reviewed the research work.

All authors read and approved the final manuscript.

## Declaration of competing interest

The authors declare that they have no competing interests.

## Data Availability

The datasets of the present study are available from the corresponding author upon request.
